# The use of phosphodiesterase inhibitors in the treatment of female sexual dysfunction: scoping review

**DOI:** 10.61622/rbgo/2024rbgo49

**Published:** 2024-07-26

**Authors:** Joana Catarina Costa Martins, Ana Rosa Cristão Afonso Lucas, Joana Margarida Meneses Costa

**Affiliations:** 1 USF São Vicente Porto Portugal Family Medicine, USF São Vicente, Porto, Portugal.; 2 USF Renascer Porto Portugal Family Medicine, USF Renascer, Porto, Portugal.; 3 USF Valbom Porto Portugal Family Medicine, USF Valbom, Porto, Portugal.

**Keywords:** Libido, Female, Sexual dysfunction, Phosphodiesterase inhibitor, Sildenafil

## Abstract

**Objective:**

The purpose of this study was to analyze the available evidence regarding the efficacy of iPDE5 in the treatment of female sexual dysfunction (FSD).

**Methods:**

A comprehensive literature search was conducted in March 2023 through the main scientific databases.

**Results:**

A total of 53 articles were identified, out of which, 6 met the predefined inclusion criteria. All of these were randomized controlled trials. Among the included studies, 4 demonstrated the effectiveness of sildenafil in improving sexual response and addressing FSD, while 2 studies failed to establish its efficacy in this context.

**Conclusion:**

Overall, the efficacy of sildenafil in the treatment of FSD remains controversial and inconclusive based on the available evidence. Further research is necessary to clarify the therapeutic potential of iPDE5 in addressing FSD and to better understand the factors that influence treatment outcomes.

## Introduction

According to the World Health Organization (WHO), health encompasses a comprehensive state of physical, psychological, and social well-being, extending beyond the mere absence of disease. This inclusive concept of health also incorporate sexual health.^(1)^ The definition of Female Sexual Dysfunction (FSD) has evolved over time and currently aligns with the International Classification of Diseases 11th Revision (ICD-11). FSD are syndromes that comprise the various ways in which an adult may have difficulty experiencing personally satisfying, non-coercive sexual activities. Sexual response is a complex interaction of psychological, interpersonal, social, cultural and physiological processes and one or more of these factors may affect any stage of the sexual response. In order to be considered a sexual dysfunction, the dysfunction must occur frequently, although it may be absent on some occasions; have been present for at least several months; and be associated with clinically significant distress. The FSD can be divided into Hypoactive Sexual Desire Dysfunction (HSDD), Sexual Arousal Dysfunction and Orgasmic Dysfunction.^(2)^

The estimated prevalence of FSD ranges from 40% to 70%, with HSDD being the most prevalent diagnosis.^(3-7)^Given its high prevalence, it is imperative to familiarize oneself with its diagnosis and stay abreast of therapeutic possibilities.

In recent years, there has been a growing interest in identifying pharmaceutical agents suitable for treating FSD. Among these, phosphodiesterase 5 inhibitors (iPDE5) serve as notable examples. While these drugs have already received approval and are in use for male erectile dysfunction, their approval for treating FSD in all countries remains pending. Nevertheless, due to their underlying mechanism of action, there is a belief that a parallel may exist between the two conditions, thus driving the interest in understanding their efficacy in women. Studies have shown that treatment with iPDE5 in women also induces relaxation of the smooth muscles in the clitoris and increases blood flow in this region. Consequently, iPDE5 were hypothesized to be a promising therapeutic option for FSD, namely sexual arousal dysfunction, however the function of the female sexuality is complex.^(8)^

The objective of this scoping review is to conduct a comprehensive analysis of the existing scientific evidence regarding the efficacy of phosphodiesterase 5 inhibitors in the treatment of female sexual dysfunction.

## Methods

A literature search was conducted in March 2023 using the keywords (“libido” OR “sexual dysfunction”) AND (“sildenafil” OR “phosphodiesterase inhibitors”) to identify relevant systematic reviews, meta-analyses, original studies, and published clinical guidelines published from the year 2000 up to March 2023 in both Portuguese and English. The following databases were utilized: National Guideline Clearinghouse, Canadian Medical Association Practice Guidelines Infobase, PubMed, The Cochrane Library, DARE, Bandolier, Evidence-Based Medicine Online, TRIP Database, and National Institute for Health and Care Excellence (NICE). The references of the included articles on the subject of the present study were also carefully reviewed to complete the search. The search results from these databases were merged and duplicates were deleted.

The inclusion criteria was women aged 18 years or older, diagnosed with female sexual dysfunction (FSD), who were administered oral phosphodiesterase 5 inhibitors (specifically sildenafil) at any dose and posology, compared to placebo. The primary focus of evaluation of improvement in FSD was through the use of validated questionnaires. Studies were excluded if the participants were concurrently receiving therapy or had an underlying pathology that could contribute to or cause sexual dysfunction. Additionally, studies involving women on medications other than phosphodiesterase 5 inhibitors for FSD treatment were excluded.

The articles included in this review were selected after careful analysis and unanimous agreement among all three authors. The selection of related articles was performed in two separate stages. In the screening phase, article titles were initially reviewed to determine inclusion. In cases of ambiguity, abstracts were examined to align with inclusion and exclusion criteria. Articles with were ambiguous and did not clearly fit the criteria proceeded to the second stage for a more careful analysis. In the second phase, the full text of articles was reviewed, and those meeting the inclusion and exclusion criteria were included in the scoping review.

To assign levels of evidence and strengths of recommendation, the Strength of Recommendation Taxonomy (SORT) scale developed by the American Academy of Family Physicians was employed.

## Results

**Figure 1:**
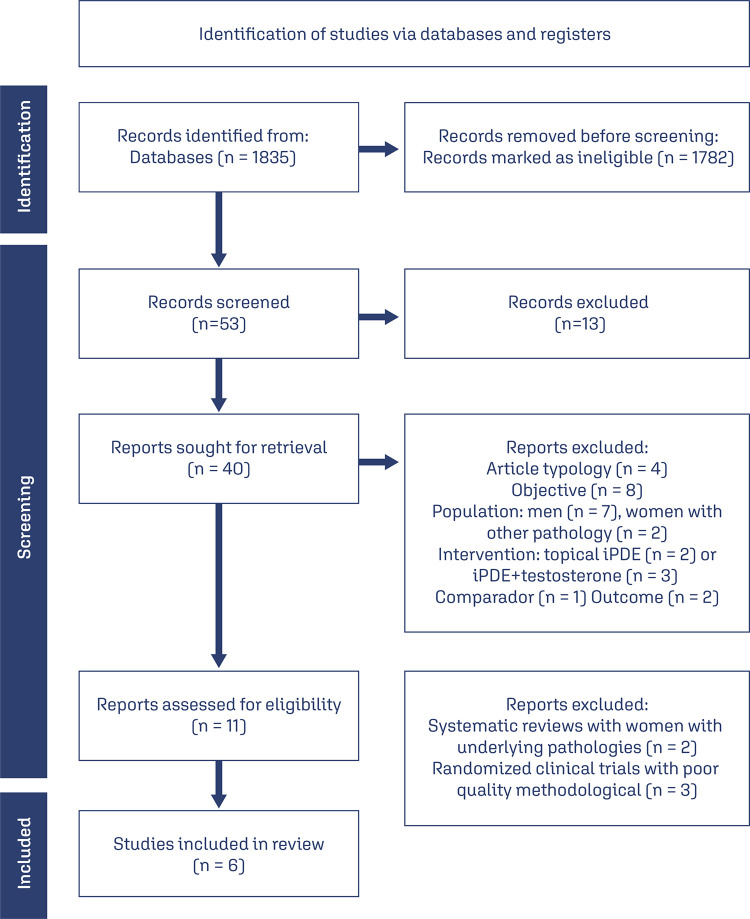
Flow chart of study selection including studies involving women with underlying pathologies that could potentially

A comprehensive search yielded a total of 1835 articles, which underwent an initial selection based on an abstract screening and subsequent full-text evaluation. Ultimately, only 6 articles met the eligibility criteria and were included in this review. The selection process of the included articles is presented in figure 1. Out of the 11 articles selected for full- text review, two systematic reviews and three randomized clinical trials were excluded. The reasons for exclusion were as follows: the two systematic reviews included articles that did not meet the predefined inclusion and exclusion criteria, influence FSD. Moreover, one of the systematic reviews only included a single relevant article of interest. The three randomized clinical trials were excluded based on concerns regarding their methodological quality.

All articles included in this review were randomized controlled trials (RCTs) of the placebo-controlled design (Chart 1), with a combined total sample size of 1188 women. The most frequently employed phosphodiesterase inhibitor was on-demand sildenafil, administered 1 hour prior to sexual intercourse. The dosages used varied among the studies, with one study utilizing a dosage of 100 mg, six studies using 50 mg, one study using 25 mg, and another study using 10 mg. Only one study employed a daily dosage of 50 mg of sildenafil. Sexual function assessment was conducted in all studies using validated questionnaires specifically designed for this purpose. However, it should be noted that the questionnaires employed were not uniform across the studies. Among the subset of five studies in which the intervention involved on- demand administration of sildenafil one hour prior to sexual activity, three studies demonstrated efficacy or observed certain benefits in the treatment of female sexual dysfunction.

**Chart 1:** Summary of the Randomized Clinical Trials included in this review

References	Population	Intervention	Outcome	Results	Conclusion	EL
Caruso et al. (2001)^(9)^ Italy	Premenopausal women diagnosed with FSAD (n=59)	Randomized to 1 of 6 sequence possibilities with the three therapeutic regimens: placebo, 25 mg sildenafil, and 50 mg sildenafil. Each therapeutic regimen was 4 weeks long, with one week break between regimens.	Personal Experience Questionnaire	Arousal and orgasm improved with sildenafil compared to placebo (p<0.001). Frequency of fantasies, frequency of sexual intercourse, satisfaction with frequency, and overall satisfaction improved with sildenafil compared to placebo (p<0.005).	Sildenafil can improve female arousal dysfunction as well as other aspects of female sexuality.	2
Basson et al. (2002)^(10)^ Canada	Premenopausal /menopausal FSAD women with HRT (n=577) or postmenopausa l women without HRT (n=204)	Sildenafil 10 mg (n=142) vs Sildenafil 50 mg (n=143) vs Sildenafil 100 mg (n=141) vs Placebo (n=151) for 12 weeks in premenopausa l/menopausal women with HRT Sildenafil 50 mg (n=103) vs Placebo (n=101) for 12 weeks in women without HRT	Two global efficacy questions, event log, LSC and SFQ at the end of 12 weeks	No statistically significant difference between sildenafil and placebo in the parameters evaluated in both premenopausal or menopausal women with HRT and postmenopausal women without HRT.	Sildenafil in women with broad spectrum FSAD showed no efficacy.	2
Basson and Brotto (2003)^(11)^ Canada	Postmenopaus al women with HRT for more than 6 months and diagnosed with FSAD (n=34)	Sildenafil 50mg session one and Placebo session two (n=17) vs Placebo session one and Sildenafil 50mg session two (n=17) Sildenafil 50mg and Placebo 1 hour prior to a session - viewing a 30 min erotic movie while using a portable vibrator with direct clitoral stimulation of standard intensity for all women	Latency to orgasm and questionnaire about the subjective intensity of orgasm and general sexual arousal	The latency to reach orgasm was 1341 seconds with placebo and 1363 seconds with sildenafil (p>0.05). The mean intensity of orgasm was 5.8 with placebo and 6.5 with sildenafil (p>0.05). Neither the main effect of sildenafil (p>0.05) nor the main effect of placebo (p>0.05) reached statistical significance for the subjective intensity of orgasm and general sexual arousal.	Sildenafil has no benefit in postmenopausal women on HRT and FSAD.	2
Berman et al . (2003)^(12)^ USA	Postmenopaus al women on HRT for more than 3 months or hysterectomize d with FSAD and with or without HSDD (n=208)	Sildenafil 50 mg (n=103) vs Placebo (n=105) for 12 weeks	SFQ and FIEI	FSAD+HSDD: no statistically significant differences between sildenafil and placebo. FSAD: sildenafil associated with improvement in all FIEI questions especially 2 - increased sensation in the genital area during intercourse (p=0.0004) and 4 - increased satisfaction with intercourse and/or foreplay (p=0.0001), as well as in the SFQ domains sensation of arousal (p<0.001), lubrication (p=0.003) and orgasm (p=0.01).	Sildenafil was associated with significant improvements in sexual arousal, orgasm, inter- course and overall satisfaction with sexual life in postmenopausal women with FSAD without concurrent HSDD who had protocol specified estradiol and free testosterone concentrations or were receiving estrogen and/or androgen replacement therapy.	2
Cavalcanti et al. (2008)^(13)^ Brazil	Postmenopaus al women diagnosed with FSD (n=22)	Sildenafil 50 mg daily (n=11) vs Placebo for 15 days (n=11)	GRISS and data on lubrication, clitoral sensitivity and orgasm	The mean GRISS value had an increase with sildenafil of 9.47% compared to baseline (p<0.05) and 3.37% compared to placebo (p<0.05). Lubrication and clitoral sensitivity increased with sildenafil compared to baseline (p<0.05) and placebo (p<0.05). The intensity of orgasm increased by 64% and 18% in the sildenafil and placebo groups (p<0.05).	Sildenafil may improve sexual response in postmenopausal women with FSD.	2
Zeinalzadeh et al. (2017)^(14)^ Iran	Premenopausal women with FSD (n=84)	Sildenafil 50 mg (n=42) vs Placebo (n=42) for 4 weeks	FSFI	The mean values of sexual desire (p=0.011), sexual arousal (p=0.001), lubrication (p=0.026), orgasm and sexual satisfaction (p<0.001) were higher with sildenafil. The mean values of sexual arousal, orgasm, sexual satisfaction and function (p<0.001) and sexual desire (p=0.001) were higher with sildenafil.	Sildenafil was effective in increasing sexual desire, arousal, orgasm and sexual satisfaction.	2

EL – Evidence level; FSD - Female Sexual Dysfunction; HRT - Hormone Replacement Therapy; LSC - Life Satisfaction Checklist; SFQ - Sexual Function Questionnaire; FSAD - Female Sexual Arousal Dysfunction; HSDD - Hypoactive Sexual Desire Dysfunction; FIEI - Female Intervention Efficacy Index; FSFI - Female Sexual Function Index; GRISS - Golombok Rust Inventory of Sexual Satisfaction

Caruso et al.^(9)^conducted an RCT with 59 premenopausal women diagnosed with FSAD. The participants were randomized to receive placebo, 25 mg of sildenafil, or 50 mg of sildenafil for 4 weeks, with a 1-week washout period between treatment phases. The women were instructed to take the medication 1 hour before sexual intercourse. The Personal Experience Questionnaire was administered before the intervention and at the end of each 4-week treatment phase, assessing levels of excitement, orgasm, frequency of fantasies and sexual intercourse, satisfaction with frequency, and overall satisfaction with sexual activity. In all assessed parameters, both 25 mg and 50 mg of sildenafil showed improvement compared to placebo, but there was no statistically significant difference between the two dosages. The authors concluded that sildenafil may improve FSAD. Two studies by Basson et al.^(10)^and Basson and Brotto^(11)^ were included in this review. In both studies, sildenafil was found to be ineffective.

In the study conducted in 2002,^(10)^a total of 781 women with FSD were divided into two groups: Group 1 included premenopausal or menopausal women on hormone replacement therapy (HRT), and Group 2 included postmenopausal women without HRT. After an initial 4-week period without treatment, the women were randomized to receive either 10 mg, 50 mg, or 100 mg of sildenafil or placebo in Group 1, while Group 2 received only 50 mg of sildenafil or placebo. At the end of the 12-week follow-up, there was no statistically significant difference between sildenafil and placebo in terms of sexual function parameters, global efficacy, life satisfaction, or sexual function questionnaire scores. Thus, sildenafil did not demonstrate efficacy in this sample of women with a broad spectrum of FSD complaints. The authors suggested conducting a study in women with FSD without HSDD, a more particular subgroup.

The study by Basson and Brotto, in 2003^(11)^included a subgroup of 34 postmenopausal women who had been on HRT for at least 6 months and had a diagnosis of FSAD with significant loss or delayed and/or decreased intensity of orgasm. These women were randomized to receive 50 mg of sildenafil or placebo in a crossover design. The intervention consisted of watching a 30-minute erotic film while using a standard intensity portable vibrator with direct clitoral stimulation. The primary outcomes assessed were orgasm latency and intensity. Subjective intensity of orgasm, awareness of genital sensations, and subjective ratings of overall sexual excitement were evaluated using self- report questionnaires. There was no statistically significant difference between sildenafil and placebo in these assessed parameters, indicating that the efficacy of sildenafil was not demonstrated in this group of women.

Berman et al.^(12)^conducted an RCT with 208 postmenopausal women on HRT or hysterectomized women with free testosterone levels >0.9 pg/ml and estradiol levels >40 pg/ml. The participants were randomized into two groups: one group received

50 mg of sildenafil 1 hour before sexual intercourse, and the other received placebo. Women with symptoms of FSAD, with or without HSDD, were recruited. The study consisted in a 4-week period without intervention, followed by a 12-week intervention phase. The primary endpoint of the study was improvement in the Female Intervention Efficacy Index (FIEI) scores for increased genital sensation during sexual intercourse and greater satisfaction with sexual intercourse and/or foreplay. All women in the sildenafil group achieved the primary endpoint compared to the placebo group (p=0.017 and p=0.015, respectively). However, statistical analysis revealed an interaction between sildenafil administration and the concomitant diagnosis of HSDD. When analyzing the results in women with FSAD and HSDD versus women with FSAD only, it was found that the former did not reach the primary endpoint, while the latter did. Therefore, the administration of 50 mg of sildenafil 1 hour before sexual activity may be effective in women with FSAD without HSDD. The authors also mentioned that the population in this study received HRT or had adequate levels of free testosterone and estradiol, as the authors believe that these factors may enhance the efficacy of sildenafil.

Cavalcanti et al.^(13)^recruited a group of 22 postmenopausal women with FSD. The participants were randomized to receive a daily dose of 50 mg of sildenafil for 15 days or placebo. This study evaluated responses to the GRISS questionnaire and clitoral artery blood flow using Doppler ultrasound. However, for the purpose of this review, only the data related to the questionnaire were analyzed. The results are presented in chart 1. The authors concluded that sildenafil may improve sexual response in postmenopausal women with orgasmic disorders.

Zeinalzadeh et al.^(14)^conducted a study with 84 premenopausal women diagnosed with FSD. The participants were randomized to receive either 50 mg of sildenafil 1 hour before sexual intercourse for 4 weeks or placebo every 12 hours for 35 days. The various parameters of the Female Sexual Function Index (FSFI) were evaluated before and after the intervention. The findings of the study showed that sildenafil was effective in increasing sexual desire, arousal, orgasm, and sexual satisfaction. However, considering the sample size of the study, further research is needed on this topic.

## Discussion

Phosphodiesterase inhibitors have demonstrated relative success in the treatment of erectile dysfunction (ED) in men. These inhibitors act by inhibiting phosphodiesterases, which are enzymes responsible for the breakdown of cyclic guanosine monophosphate (cGMP) and cyclic adenosine monophosphate (cAMP), intracellular messengers that mediate hormone and neurotransmitter responses. Among the various phosphodiesterases, phosphodiesterase 5 (PDE5) is the target for ED treatment due to its location in the corpus cavernosum, leading to vasodilation and increased blood flow.^(8,15)^ A similar mechanism occurs in women: relaxation of the smooth muscle of the vagina and clitoris and increased blood flow to these organs is one of the essential phase of the female sexual response and results in increased vaginal lubrication, engorgement of the walls and luminal diameter, as well as an increase in the length and diameter of the clitoris. Although this biological effect exists, it remains unproven whether it translates into increased and improved female sexual response, given the complex and multifactorial nature of FSD.^(8)^

The present review aimed to analyze the available scientific evidence on the efficacy of using iPDE5 in the treatment of FSD. In this review, four studies demonstrated improvement in certain parameters with the use of iPDE5 in FSD, while two studies failed to establish its efficacy. Although all of these studies were randomized and placebo- controlled, with well-established methodologies, they suffered from small sample sizes and short follow-up periods. Three of the studies^(10-12)^received support from Pfizer. However, they were included in the review due to their rigorous methodology, and only one of them demonstrated a positive effect of the drug.

The total sample size across the studies was 1188 women, reflecting a substantial number of participants. Among the subset of five studies in which the intervention involved on-demand administration of sildenafil one hour prior to sexual activity, three studies demonstrated efficacy or observed certain benefits in the treatment of FSD. This finding suggests a potential positive effect of sildenafil in improving sexual function and addressing FSD in women.

These findings suggest that sildenafil may have a positive impact on sexual function in women with FSD. Notably, the positive outcomes were observed in various domains of sexual function, including increased genital sensation during sexual intercourse, greater satisfaction with sexual intercourse and/or foreplay, improved sexual desire, arousal, orgasm, and sexual satisfaction. The improvement in these parameters is in the line with the mechanism of iPDE5 and the female sexual response.

The participants in these studies were generally healthy women, but the samples varied, including premenopausal and postmenopausal women with or without hormone replacement therapy (HRT), leading to heterogeneity within the study population. It is important to recognize that a woman’s sexual function changes throughout her life and is influenced by various factors. Therefore, these factors may introduce limitations when interpreting the results, particularly considering that individual, relational, and cultural factors shape the experience of sexuality, especially considering that the populations across the different studies were from different countries with diverse cultural backgrounds.

Another limitation pertains to the broad definition and diagnosis of FSD, which, despite being well-established, still engenders controversy regarding the differentiation of various concepts within this overarching diagnosis. This limitation is evident in certain articles where differences in drug efficacy were observed among women presenting different complaints and symptoms along the spectrum of FSD.

Most studies administered the drug on-demand, while only one study used a daily dosage regimen. There was a lack of consensus regarding the most effective dosage, further complicating the interpretation of the results. Additionally, we chose not to include articles that investigated the concurrent use of iPDE5 and testosterone, as the scope of this review was solely to evaluate the efficacy of iPDE5.

Lastly, it is worth noting that the assessment of sexual function and the various instruments used to measure outcomes were also characterized by heterogeneity across the studies, posing an additional challenge in the interpretation of results.

## Conclusion

This scoping review evaluated the available literature on the use of iPDE5 in the treatment of FSD. The search process yielded a substantial number of articles, with a rigorous selection procedure resulting in the inclusion of six RCT in this review. Among the selected studies, the majority employed on-demand administration of sildenafil, one hour prior to sexual activity. The dosages of iPDE5 varied across the studies, with sildenafil being the most commonly used drug. Notably, three out of the five studies focusing on on-demand sildenafil demonstrated efficacy or observed some beneficial effects in the treatment of FSD. However, the heterogeneity of the study populations, encompassing both premenopausal and postmenopausal women with or without hormone replacement therapy, may have influenced the interpretation of results. The complex and multifactorial nature of FSD, influenced by individual, relational, and cultural factors, adds further complexity to the assessment of treatment outcomes. In conclusion, this review contributes with valuable insights into the efficacy of iPDE5 in the context of FSD (SORT B). Further research with larger sample sizes, longer follow-up periods, standardized assessment tools, and awareness of diverse cultural backgrounds is essential to enhance the understanding of the role of iPDE5 in the treatment of FSD.
